# The Structure of *Plasmodium falciparum* Blood-Stage 6-Cys Protein *Pf*41 Reveals an Unexpected Intra-Domain Insertion Required for *Pf*12 Coordination

**DOI:** 10.1371/journal.pone.0139407

**Published:** 2015-09-28

**Authors:** Michelle L. Parker, Fangni Peng, Martin J. Boulanger

**Affiliations:** Department of Biochemistry & Microbiology, University of Victoria, Victoria, British Columbia, Canada; Tulane University, UNITED STATES

## Abstract

*Plasmodium falciparum* is an apicomplexan parasite and the etiological agent of severe human malaria. The complex *P*. *falciparum* life cycle is supported by a diverse repertoire of surface proteins including the family of 6-Cys s48/45 antigens. Of these, *Pf*41 is localized to the surface of the blood-stage merozoite through its interaction with the glycophosphatidylinositol-anchored *Pf*12. Our recent structural characterization of *Pf*12 revealed two juxtaposed 6-Cys domains (D1 and D2). *Pf*41, however, contains an additional segment of 120 residues predicted to form a large spacer separating its two 6-Cys domains. To gain insight into the assembly mechanism and overall architecture of the *Pf*12-*Pf*41 complex, we first determined the 2.45 Å resolution crystal structure of *Pf*41 using zinc single-wavelength anomalous dispersion. Structural analysis revealed an unexpected domain organization where the *Pf*41 6-Cys domains are, in fact, intimately associated and the additional residues instead map predominately to an inserted domain-like region (ID) located between two β-strands in D1. Notably, the ID is largely proteolyzed in the final structure suggesting inherent flexibility. To assess the contribution of the ID to complex formation, we engineered a form of *Pf*41 where the ID was replaced by a short glycine-serine linker and showed by isothermal titration calorimetry that binding to *Pf*12 was abrogated. Finally, protease protection assays showed that the proteolytic susceptibility of the ID was significantly reduced in the complex, consistent with the *Pf*41 ID directly engaging *Pf*12. Collectively, these data establish the architectural organization of *Pf*41 and define an essential role for the *Pf*41 ID in promoting assembly of the *Pf*12-*Pf*41 heterodimeric complex.

## Introduction

Apicomplexan parasites of the *Plasmodium* genus are the etiological agents of malaria, one of the most severe and widespread infectious diseases in the developing world. While several species of *Plasmodium* can cause malaria in humans, *P*. *falciparum* is the most lethal [1]. In 2013, there were an estimated 198 million cases of malaria that led to 584,000 deaths, 90% of which occurred in Africa and the majority of which were caused by *P*. *falciparum* infection [1]. The success of *Plasmodium* spp. is due, in part, to a complex life cycle that relies on sophisticated molecular strategies to both survive transmission in mosquito vectors and access the immuno-protective environment of host cells [2, 3]; to accomplish this, *Plasmodium* parasites encode a diverse arsenal of surface displayed proteins capable of interfacing with biomolecular partners on vector and host cells. The 6-Cys s48/45 family of surface antigens has garnered particular attention as they are differentially expressed at every stage of the *P*. *falciparum* life cycle and have been shown to play important biological roles [4].

Of the 14 members that comprise the 6-Cys s48/45 protein family in *Plasmodium falciparum* [5, 6], *Pf*36, *Pf*52, sequestrin and B9 are expressed on the sporozoite or liver-stage parasite surface and play critical roles in invasion of or growth within hepatocytes [6–9]. *Pf*s230, *Pf*s48/45 and *Pf*s47, designated with the “s” for sexual stage specific antigens, are presented on the surface of gametocytes and are involved in fertilization of male and female gametes in the human blood stream as well as escape from the mosquito immune system [10–12]. *Pf*12, *Pf*38, *Pf*41 and *Pf*92 are localized on the surface of the blood-stage merozoite, which is the form of the parasite that invades erythrocytes, proliferates, and causes the symptoms of malaria [2]. On the merozoite surface, *Pf*12 and *Pf*41 are displayed as a heterodimer with *Pf*12 tethered to the outer membrane via a glycophosphatidylinositol (GPI) anchor and soluble *Pf*41 appropriately localized through its interaction with *Pf*12 [13–16]. Notably, *Pf*12 is the fifth most prevalent GPI-anchored protein on the merozoite surface [13], both *Pf*12 and *Pf*41 are strongly recognized by antibodies from naturally infected patients [16–19], and *Pf*41 was recently identified as one of five top-ranked potential malaria vaccine candidates [20]. Strikingly, however, no phenotype was observed in *Pf*12 or *Pf*41 knockout parasites, although it was noted that this observation may be due to the ability of the parasites to adapt in culture through activating compensatory mechanisms [14].

**Fig 1:**
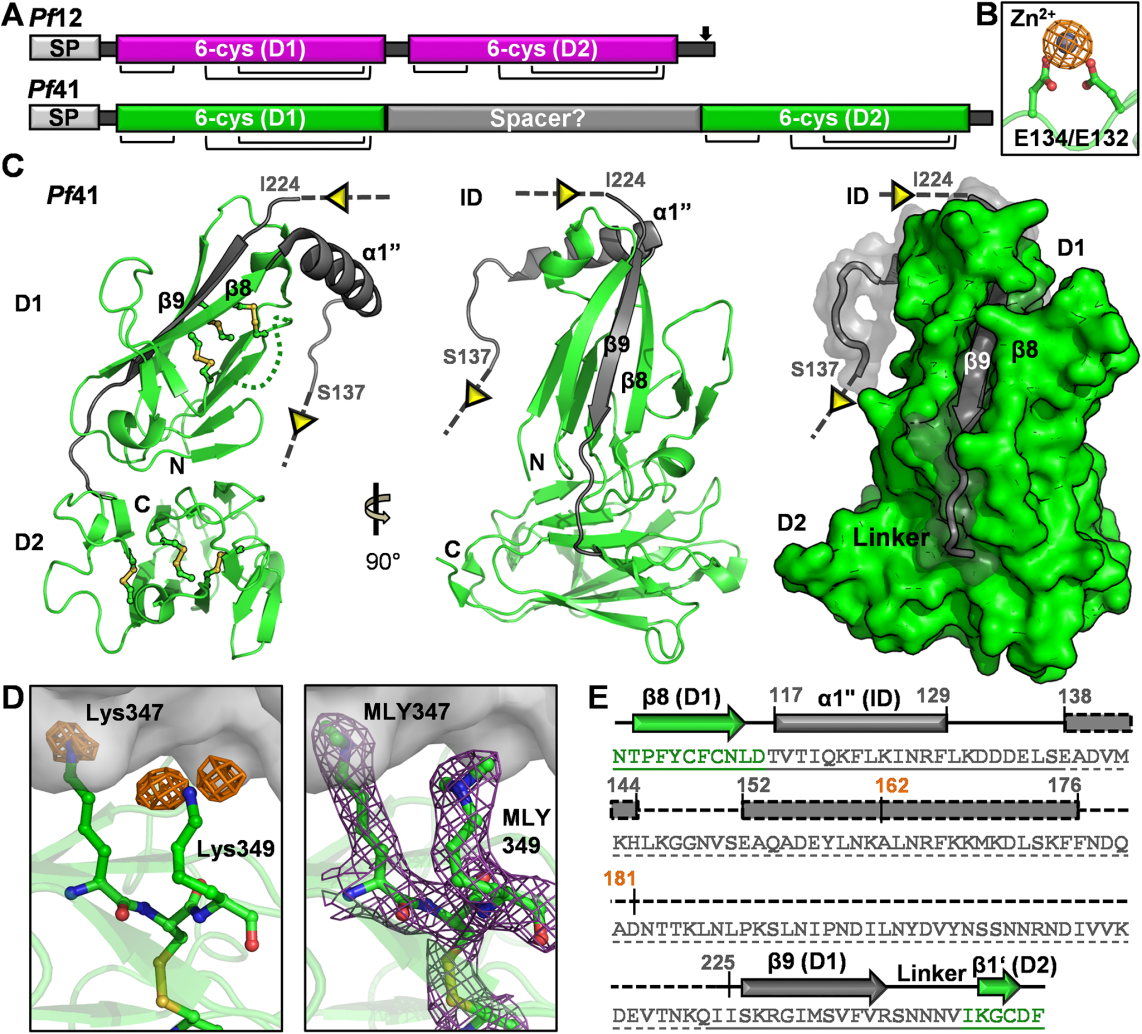
The structure of *Pf*41 reveals that a large sequence insertion predominately maps between two β-strands in D1. **(A)** Schematic comparison of predicted *Pf*41 domain organization with established *Pf*12 tandem 6-Cys domains. SP, signal peptide; D1, domain 1; D2, domain 2; arrow indicates GPI anchor attachment site. Black bars indicate disulfide connectivity. **(B)** Anomalous difference electron density map of *Pf*41 calculated at 8 sigma (orange mesh) around one of two high confidence zinc sites (grey sphere) used for phasing. Two Glu residues (Glu 132 and 134) were found to coordinate the zinc ion (along with His23 and Glu369 of two neighboring chains, not shown) and are shown in green ball-and-stick colored by element. **(C)** Left/middle**–**Orthogonal views of the *Pf*41 structure, shown as a cartoon backbone colored as in (A). Disulfides are shown in the left panel as ball-and-stick colored by element. A four residue un-modeled region in D1 is indicated by a dotted green connecting loop. Grey dotted lines extending out of α1” (inserted domain-like region, ID) and into β9 (D1) indicate the un-modeled region of the ID (~85 residues). Right–green surface of *Pf*41 in same orientation as (C, middle) colored as in (A), with the previously undefined region shown with a semi-transparent grey surface with underlying cartoon. **(D)** Left–Lys349 shown as green sticks colored by element with the positive Fo-Fc map shown as an orange mesh contoured at 2.5σ. Right–final σ-A weighted 2Fo-Fc electron density map shown as a purple mesh contoured at 1σ around the dimethylated lysines (MLY347 and 349). The symmetry mate against which the methylated lysines pack is shown as a semi-transparent grey surface. **(E)** Sequence of the ID and flanking secondary structure elements colored as in (A). Regions observed in the structure have a solid underline; un-modelled sequence and predicted secondary structure elements are indicated by dashed lines.

Recent structural analysis of *Pf*12 provided the first insight into the architecture of the 6-Cys domain and the organization of the tandem repeats with the two disulfide pinned β-sandwich domains connected via a short linker [5, 15]. Intriguingly, *Pf*41 incorporates an additional 120 residues of unknown structure that appear to form a spacer linking the two predicted 6-Cys domains (D1 and D2) (Fig 1A). Based on the expected domain organization of *Pf*41 [17], the structure of *Pf*12 [15], and chemical cross-linking data [15], we previously proposed a preliminary model where *Pf*12 and *Pf*41 adopt an antiparallel organization [15]. A weakness of this model, however, is the absence of structural information describing *Pf*41 and, in particular, the large sequence insertion. To address this limitation, we report here the crystal structure of *Pf*41 and complement the structural data with solution binding studies to reveal the molecular determinants of *Pf*12-*Pf*41 complex formation. Based on these data, we re-evaluate and refine the model of the assembly mechanism and overall architecture of the *Pf*12-*Pf*41 heterodimer.

## Materials and Methods

### Cloning, protein production and purification

Two forms of *Pf*41 and one form of *Pf*12 were recombinantly produced in this study. The mature full length *Pf*41 construct extends from the predicted signal peptide cleavage site to the C-terminus (Lys21 to Ser378; PlasmoDB: PF3D7_0404900), while the *Pf*12 construct extends from the signal peptide cleavage site to the GPI anchor site (His26 to Ser321; PlasmoDB: PF3D7_0612700) as described previously [15]. For both *Pf*12 and *Pf*41, N-linked glycosylation sites were mutated and the genes codon optimized for insect cells, synthesized and subcloned into a modified pAcSecG2T vector (Pharmingen) with a TEV protease cleavable N-terminal hexahistidine/maltose binding protein (MBP) tag. The *Pf*41 construct lacking the inserted domain-like region (*Pf*41ΔID) was cloned out of *Pf*41 by overlap extension PCR enabling the replacement of the ID (Thr117 to Ile225) with a GSGGSG linker. Protein production and purification was performed using established protocols [15]. Briefly, expression viruses were generated and amplified in *Spodoptera frugiperda* 9 cells and protein production was performed in Hi5 cells. Growth media was harvested after a 65 hour infection and secreted proteins purified by nickel affinity chromatography. The hexahistidine/MBP tag was cleaved with TEV protease and removed by cation exchange chromatography. Proteins were further purified by size exclusion chromatography (SEC) in HEPES-buffered saline (HBS: HEPES pH 7.5, 150mM NaCl) with 2% glycerol.

### Protein methylation

Prior to crystallization, *Pf*41-MBP fusion protein was concentrated to 1.4 mg/mL and dialyzed into 50 mM HEPES pH 7.5, 250 mM NaCl and 2% glycerol for lysine methylation based on a previously published protocol [21]. Briefly, borane-dimethylamine complex (Acros Organics) and methanol-free formaldehyde (Thermo Scientific) were sequentially added to final concentrations of 50 mM and 80 mM, respectively, and incubated with the protein overnight at 4°C. The methylation reaction was stopped by addition of 100 mM glycine. Methylated *Pf*41-MBP was cleaved with TEV protease and purified by cation exchange and SEC as described above. Methylated protein was used only for crystallization experiments.

### Crystallization and data collection

Crystallization trials were set using a Crystal Gryphon (Art Robbins Instruments). Crystals of methylated *Pf*41 were identified in the PACT Premier Screen (Molecular Dimensions) using sitting drops at 18°C. The final drops consisted of 0.3 μL *Pf*41 at 15 mg/mL with 0.2 μL of reservoir solution (10 mM zinc chloride, 0.1 M MES buffer pH 6.0, 20% PEG6000) and were equilibrated against 55 μL of reservoir solution. Crystals were cryoprotected in mother liquor with 15% glycerol and flash cooled in liquid nitrogen. Diffraction data were collected on beamline 12–2 at the Stanford Synchrotron Radiation Lightsource.

### Data processing, structure determination and refinement

Diffraction data were initially processed to 2.61 Å resolution using Imosflm [22] and Aimless [23]. The structure of *Pf*41 was determined by zinc single-wavelength anomalous dispersion. Two high confidence Zn sites were identified and refined using Phenix.autosol [24, 25], which enabled building of approximately 90% of the backbone using Phenix.autobuild [26]. The nearly complete model was improved following refinement in Phenix.refine [27] against the higher resolution (2.45 Å resolution) data set. Final model building and solvent atom selection was performed in COOT [28]. Structural validation was performed with MolProbity [29]. Overall, 5% of reflections were set aside for calculation of R_free_. Data collection and refinement statistics are presented in Table 1. The atomic coordinates and structure factors for *Pf*41 have been deposited in the Protein Data Bank under the following PDB ID: 4YS4.

**Table 1:** DATA COLLECTION AND REFINEMENT STATISTICS.

	*Pf*41_Zn	*Pf*41
A. Data collection statistics		
Spacegroup	I23	I23
a = b = c (Å)	133.6	133.8
α = β = γ (deg.)	90	90
Wavelength	0.9795	0.9795
Resolution range (Å)	38.58–2.61(2.72–2.61)^a^	47.29–2.45 (2.57–2.45)
Measured reflections	203,542 (21,697)	148,728 (11,834)
Unique reflections	12,258 (1,461)	14,643 (1,811)
Redundancy	16.6 (14.9)	10.2 (6.5)
Completeness (%)	99.9 (99.0)	99.0 (92.8)
*I/σ(I)*	30.3 (5.3)	18.2 (3.0)
R_merge_	0.073 (0.527)	0.078 (0.573)
Wilson B (Å^2^)	63.6	44.7
Phasing power (FOM^b^)	0.30	N/A
B. Refinement statistics		
Resolution (Å)		35.75–2.45
R_work_ / R_free_		0.182/0.228
No. of atoms		
Protein		2087
Solvent		54
Zn/Cl/Glycerol		3/2/6
Average B-values (Å^2^)		
Protein		49.5
Solvent		48.0
Zn/Cl/Glycerol		63.4/42.4/73.8
r.m.s. deviation from ideality		
Bond lengths (Å)		0.003
Bond angles (deg.)		0.746
Ramachandran statistics (%)		
Most favoured		98.0
Allowed		2.0
Disallowed		0.0

^a^ Values in parentheses are for the highest resolution shell

^b^ FOM is Figure of Merit from Phenix AutoSol [25]

### Isothermal titration calorimetry

ITC measurements were carried out at 25°C on a MicroCal iTC200 instrument (Malvern). The sample cell contained *Pf*12 at a concentration of 10 μM, with full length *Pf*41 or *Pf*41ΔID at a concentration of 100 μM added in 16 injections of 2.4 μL each separated by 180 seconds. Data were analyzed with Origin software (MicroCal) and the dissociation constant (K_d_) was determined using a one-site model. Values were derived from a single experiment, but are representative of two independent experiments.

### Trypsin protection assay

*Pf*41 was incubated with an equal molar ratio of *Pf*12 at 4°C for 10 min to allow for complex assembly. Trypsin was added to the protein samples (*Pf*12, *Pf*41, or *Pf*12-*Pf*41 mixture) at a trypsin:protein ratio of 1:100 (w/w). Reactions were incubated at 4°C. Aliquots of each reaction were taken at time intervals between 0 min and 2 hours and inactivated by the addition of protease inhibitor in SDS loading buffer. Aliquots were heated at 95°C, separated by SDS-PAGE, and visualized with Coomassie Brilliant Blue. Select protein bands were cut out of the polyacrylamide gel, trypsin digested, and analyzed by MALDI-TOF mass spectrometry as described previously [15].

## Results

### Organization of the *Pf*41 6-Cys domains reveals an unexpected juxtaposition

To investigate the domain organization of *Pf*41, including the architecture of the predicted spacer region (Fig 1A), we adopted an insect cell based expression system to produce sufficient quantities of correctly folded recombinant protein to support structural and biophysical studies [15]. *Pf*41 initially proved recalcitrant to crystallization potentially due to the high surface entropy resulting from 40 lysine residues present in the mature protein, representing approximately 11% of the total sequence. To compensate for this innate property of *Pf*41, surface lysines were chemically methylated during purification, and crystals of methylated *Pf*41 were observed after eight months. Intriguingly, attempts to solve the structure of *Pf*41 by molecular replacement were unsuccessful, likely indicating architectural divergence from the only other structurally characterized 6-Cys protein, *Pf*12 [5, 15]. Ultimately, the structure of *Pf*41 was determined by zinc single-wavelength anomalous dispersion to a resolution of 2.45 Å (Fig 1B). One *Pf*41 molecule was present in the asymmetric unit, and PISA analysis [30] indicated that *Pf*41 crystallized as a monomer consistent with a previous analysis showing no homo-multimerization capabilities [14]. The refined model of *Pf*41 begins at Lys21 and extends through Glu369, with a four residue disordered region (Lys59 to Ser62) and an extended, un-modelled region between Ser137 and Ile224 (Fig 1C, left). During refinement, two clear positive difference density peaks were observed near the epsilon amino groups of each of three lysine residues consistent with dimethyl modifications (Fig 1D, left). Two of the dimethylated lysines were located at a crystal packing interface (Fig 1D, right), underscoring the importance of lysine methylation in obtaining diffraction quality crystals of *Pf*41.

**Fig 2:**
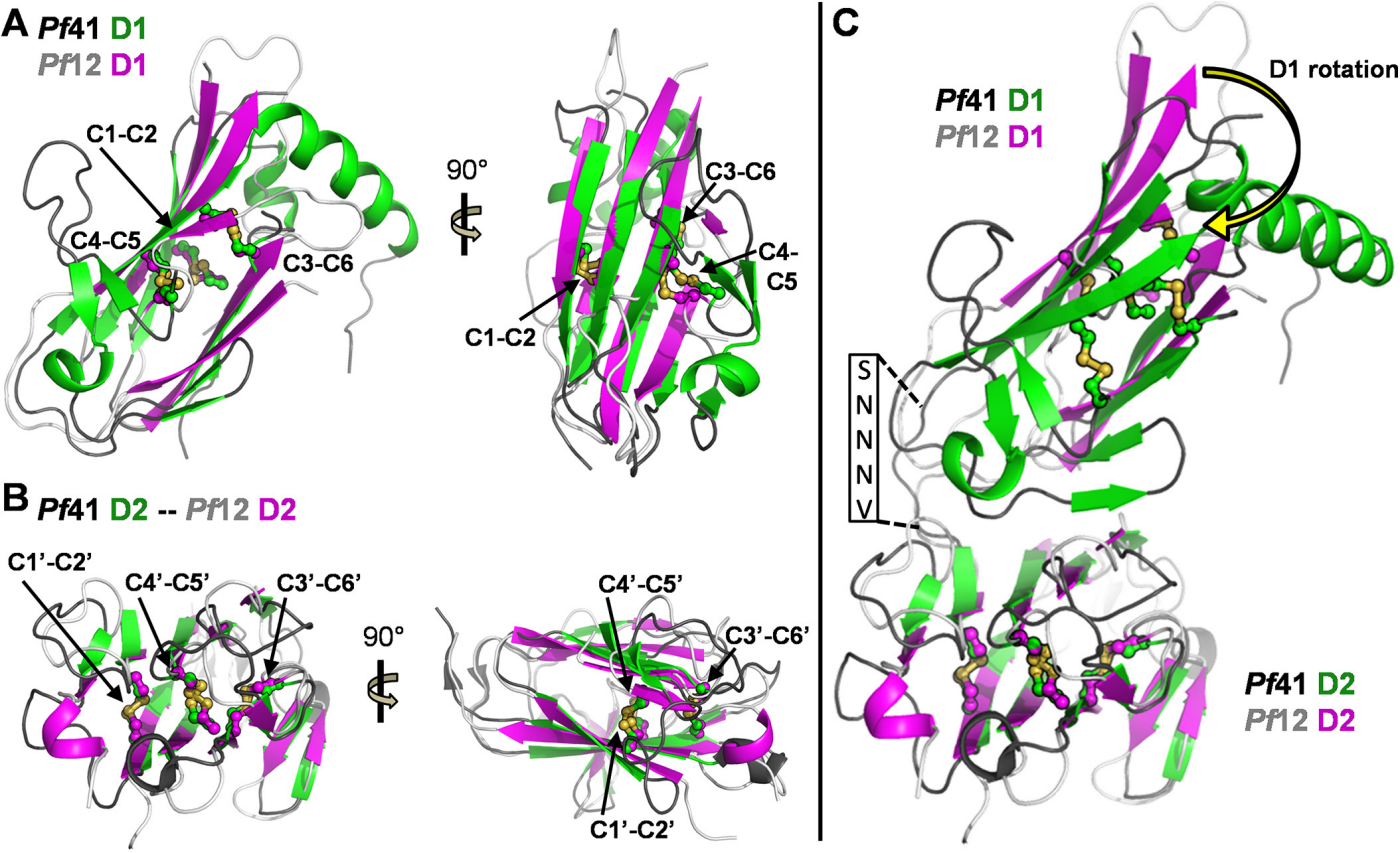
The structure of *Pf*41 refines the description of the 6-Cys domain and shows strong similarity to *Pf*12. **(A)** Orthogonal views of an overlay of *Pf*41 D1 (green cartoon with dark grey loops) on *Pf*12 D1 (PDB ID 2YMO; magenta cartoon and light grey loops). Disulfides are labeled, shown as ball-and-stick, and colored by element. **(B)** Orthogonal views of an overlay of *Pf*41 D2 on *Pf*12 D2, colored as in (A). **(C)** Overlay of *Pf*41 on *Pf*12, with the alignment anchored on D2, showing the different rotation of D1 relative to D2 (yellow curved arrow) in the two structures. The *Pf*41 linker sequence is indicated in the black box.

Structural analysis confirms that both *Pf*41 6-Cys domains (D1 and D2) adopt a β-sandwich fold with expected disulfide connectivity of C1-C2, C3-C6 and C4-C5; the first two disulfides pin together the two sheets of the β-sandwich and the latter disulfide anchors an ancillary loop to the domain core (Figs 1C and 2). As evident from structural overlays, the architecture of the two *Pf*41 6-Cys s48/45 domains are similar to those of *Pf*12 (Fig 2) [5, 15]. Specifically, the D1 domains overlay with a root mean square deviation (rmsd) of 1.1 Å over just 44 Cα atoms indicating conservation of the small domain core but divergent conformations of the inter-strand loops (Fig 2A), which is consistent with our inability to solve the structure of *Pf*41 by molecular replacement. The D2 domains were more similar as they overlay with an rmsd of 0.99 Å over 86 Cα atoms, indicating conservation of both the domain core and most inter-strand loops (Fig 2B). Intriguingly, despite a rotation of D1 relative to D2, the relative spatial positioning of the two 6-Cys domains of *Pf*41 is analogous to that of *Pf*12 (Fig 2C), with overall dimensions for these two domains of approximately 65 by 40 Å, despite the prediction that the 6-Cys domains of *Pf*41 are separated by a large spacer (Fig 1A). The unexpected topological similarity is mediated by a relatively conserved, at least with respect to sequence length, inter-domain linker in *Pf*41 comprising residues SNNNV (Fig 2C) (*Pf*12 linker sequence: SLENK [15]).

As only the second 6-Cys protein to be structurally characterized, *Pf*41 confirms that the overall architecture of the *Plasmodium* 6-Cys domains is reminiscent of the SRS fold from related Apicomplexa such as *Toxoplasma gondii* [4, 31–33]. However, one key difference is the placement of the sixth cysteine residue, which has important implications for the bioinformatic assignment of C-terminal domain boundaries: C6 of the SRS fold is located in the final β-strand resulting in a clear delinieation of the domain boundaries, whereas both C5 and C6 of the 6-Cys fold are located in the penultimate β-strand. Furthermore, there is no sequence motif defining the final inter-strand loop or β-strand of the 6-Cys fold [6], which has led to ambiguity in the definition of the C-terminal domain boundaries for members of the 6-Cys family.

### Structural analysis reveals that the large sequence insertion in *Pf*41 is not an inter-domain spacer, but is inserted within D1

To further investigate the nature of the 120 residue sequence insertion in *Pf*41 (Thr117 to Val241) relative to *Pf*12, we first sought to establish whether the lack of electron density between Ser137 and Ile224 was due to flexibility within the crystal or due to the region being proteolytically processed. Analysis of the crystal packing revealed insufficient room in the crystal lattice to accommodate the un-modelled residues leading us to conclude that the region was proteolyzed during the extended time frame in which it took the crystals to form. While a significant portion of the *Pf*41 sequence insertion residues are un-modelled in the structure, it is clear that the labile region is inserted between the last two strands of the D1 domain: β8 and β9. Altogether, 108 residues of the *Pf*41 sequence insertion lie between D1 β8 and β9 (the remaining inserted residues complete β9 and extend through the short inter-domain linker), with the modelled residues extending from β8 into an alpha helix (α1”: Thr117 to Phe129) followed by a region of coil (Leu130 to Ser137) ordered by crystal packing (Fig 1C and 1E). Based on the region observed in the crystal structure combined with secondary structure and disorder predictions [34, 35], the region between β8 and β9 is expected to contain approximately 40% α-helical character, with the presence of the longest putative helix partially supported by previous circular dichroism analysis of a component peptide (Ala162 to Asp181, Fig 1E) showing predominant helical content [36]. The remaining 60% of this region, predominately mapping to the C-terminal portion, is predicted to be present as disordered, random coil (Fig 1E). Since much of the sequence comprising the D1 β8-β9 loop is predicted to be disordered, this region could be classified as intrinsically disordered [37, 38]. However, such a designation will require validation by other methods such as nuclear magnetic resonance spectroscopy. Thus, we have termed the *Pf*41 β8-β9 loop (Thr117 to Ile225) an inserted domain-like region (ID).

Multiple sequence alignments of the *Pf*41 ID with other 6-Cys proteins anchored by the last Cys of D1 (C6 in β8) and the first Cys of D2 (C1’ in β1’) combined with secondary structure predictions clearly showed that the α-helical/coil composition of the ID is unique to *Pf*41 (data not shown). It is worth noting, however, that the related sexual stage 6-Cys protein *Pf*s47 has a predicted inter-domain region of approximately 100 residues with two cysteines and predominant β-structure that has the potential to be a well-ordered inserted domain in the analogous loop of D1. Altogether, our data reveal that the majority of the inserted sequence in *Pf*41 maps to an ID embedded in D1, and since this region appears to be unique to *Pf*41, we reasoned that its corresponding function is similarly unique. This led us to postulate a potential role for the *Pf*41 ID in coordinating *Pf*12.

### ITC data reveal that the *Pf*41 ID is necessary to coordinate *Pf*12

**Fig 3:**
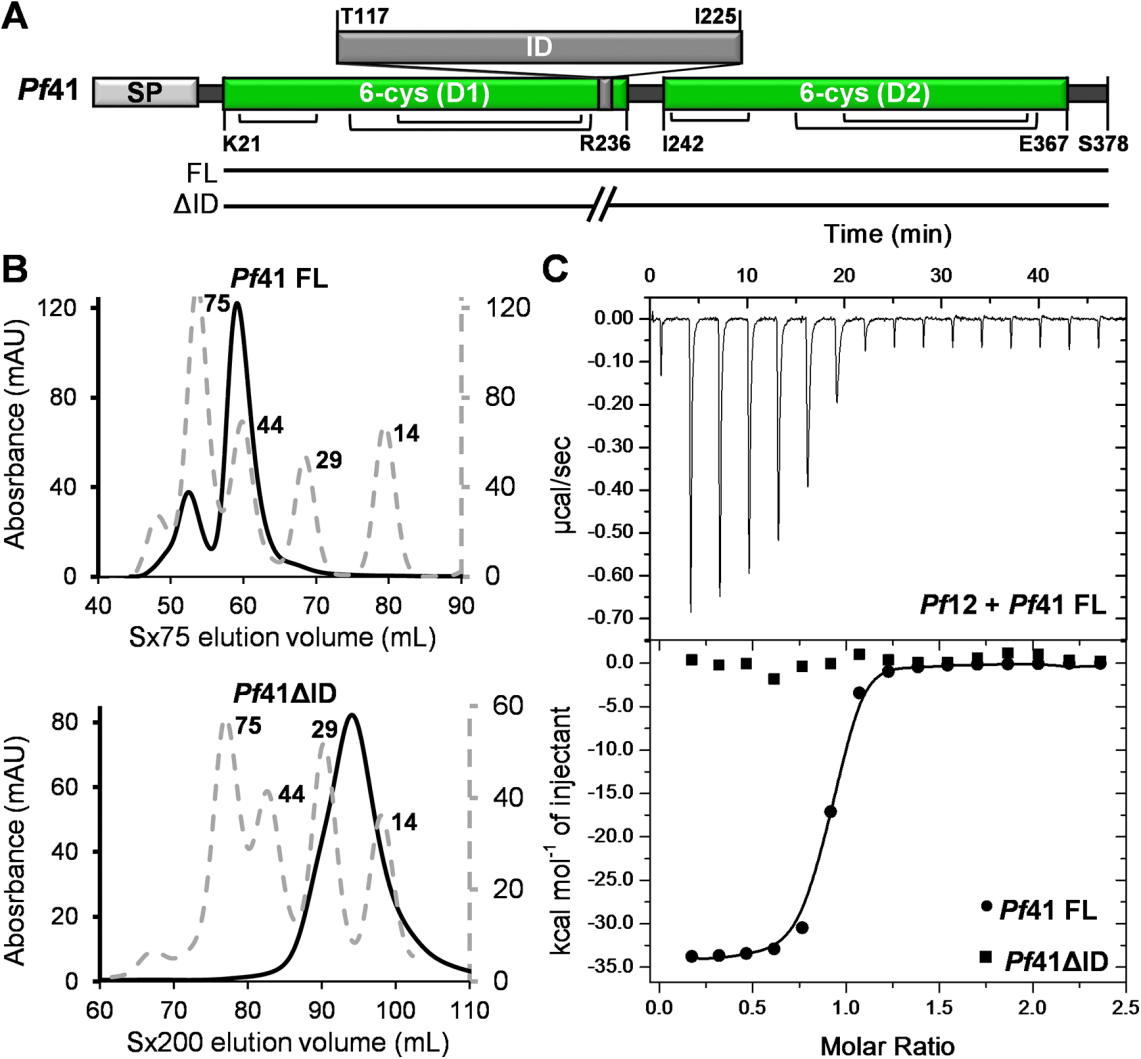
ITC analysis of *Pf*12-*Pf*41 coordination reveals a critical role for the *Pf*41 ID. **(A)** Schematic of the refined model of *Pf*41 domain organization. SP, signal peptide; D1, domain 1; D2, domain 2; ID, inserted domain-like region. Two constructs, *Pf*41 full length (FL, Lys21 to Ser378) and *Pf*41ΔID (Lys21 to Asp116–GSGGSG–Ser226 to Ser378), were used for ITC studies. **(B)** SEC column elution profiles of *Pf*41 FL (top) and *Pf*41ΔID (bottom). Solid lines represent the trace of *Pf*41 constructs (expected molecular mass: *Pf*41 FL, 41 kDa; *Pf*41ΔID, 29 kDa); the gray dashed lines represent SEC globular molecular mass standards, labelled in kDa. Note that the small peak at approximately 80 kDa for the *Pf*41 FL trace represents contaminating MBP-tagged protein due to incomplete cleavage with TEV protease. Monomeric peak fractions were pooled for ITC studies. **(C)** ITC profile of *Pf*41 constructs (FL and ΔID) titrated against *Pf*12 at 25°C.

To assess the contribution of the *Pf*41 ID to coordinating *Pf*12, we first used the ID boundaries defined from our structure to engineer a form of *Pf*41 where the ID was strategically replaced with a short glycine-serine linker (P*f*41ΔID; Fig 3A). Following proteolytic removal of the purification hexahistidine/MBP tags, both recombinant full length *Pf*41 and *Pf*41ΔID eluted from the SEC column consistent with the expected molecular weights (Fig 3B) indicating that truncation of the ID did not alter protein folding. It is important to note that the exceptionally stable disulfide-pinned core of the 6-Cys/SRS domain is commonly accessorized with loops of different lengths and compositions [4, 15, 31–33], supporting our observation that interchanging the ID sequence with a shortened loop does not disrupt the core 6-Cys structure. Moreover, we suspect that truncation of the ID does not appreciably increase inter-domain mobility, as we were able to obtain crystals of the tandem *Pf*41 6-Cys domains despite proteolytic removal of a large portion of the ID. Having established the quality of our full length and ID truncated *Pf*41, we next investigated the solution binding characteristics of these constructs with *Pf*12. Titration of *Pf*41 into the ITC cell containing *Pf*12 produced a dissociation constant (K_d_) of 27.7 ± 3.7 nM (Fig 3C), which reflects approximately one order of magnitude tighter binding than previously obtained by surface plasmon resonance using rat CD4d3/4 fused *Pf*12 and *Pf*41 constructs [14]; the difference in measured affinity likely arises from a combination of the different techniques used and the presence/absence of a fusion partner. ITC data were fitted using a single-site binding model and showed a stoichiometry of approximately 1:1 (0.85 ± 0.01 *Pf*41:*Pf*12), consistent with a *Pf*12-*Pf*41 heterodimer shown previously by SEC co-elution volume and cross-linking of both recombinant and parasite-surface proteins [14, 15]. The strongly negative binding enthalpy (ΔH of -33.9 ± 0.2 kcal/mol) indicates formation of numerous favorable interactions between full length *Pf*41 and *Pf*12, which compensate for a highly unfavorable entropy change (-TΔS of 23.6 kcal/mol). The thermodynamic parameters for the *Pf*12-*Pf*41 interaction are more extreme than typical protein-protein interactions and approach the values for interactions that require significant conformational changes [39], which may indicate that the interaction requires organization of the predicted disordered regions of the *Pf*41 ID and/or the shorter flexible loops on *Pf*12. In contrast to the tight interaction measured for the *Pf*12-*Pf*41 complex, no binding was observed between the ID truncated form of *Pf*41 and *Pf*12 indicating that the *Pf*41 ID is necessary for coordinating *Pf*12 (Fig 3C, bottom).

Having established that the absence of the *Pf*41 ID in the context of the tandem 6-Cys domains essentially eliminated binding to *Pf*12, we next sought to determine if the *Pf*41 ID was sufficient for *Pf*12 recognition. In support of this approach we engineered constructs where the ID was fused to either MBP or protein G B1 domain. We also engineered a form of the ID with cysteines at each end to better mimic the structural constraints observed in the crystal structure, where the N- and C-termini of the ID are separated by only 6.5 Å. Unfortunately, production of these fusion constructs in insect cells or *E*. *coli* did not yield a stable protein. To assess whether sufficient stability of the ID could be imparted by the inclusion of *Pf*41 D1, we engineered an MBP fusion construct extending from Lys21 through Arg236. While the incorporation of D1 markedly improved solubility and stability of the ID, removal of the MBP tag led to significant protein precipitation. Thus, it appears that both *Pf*41 D1 and D2 are necessary to stabilize the ID.

### The *Pf*41 ID becomes protected from proteolysis in the *Pf*12-*Pf*41 heterodimer

**Fig 4:**
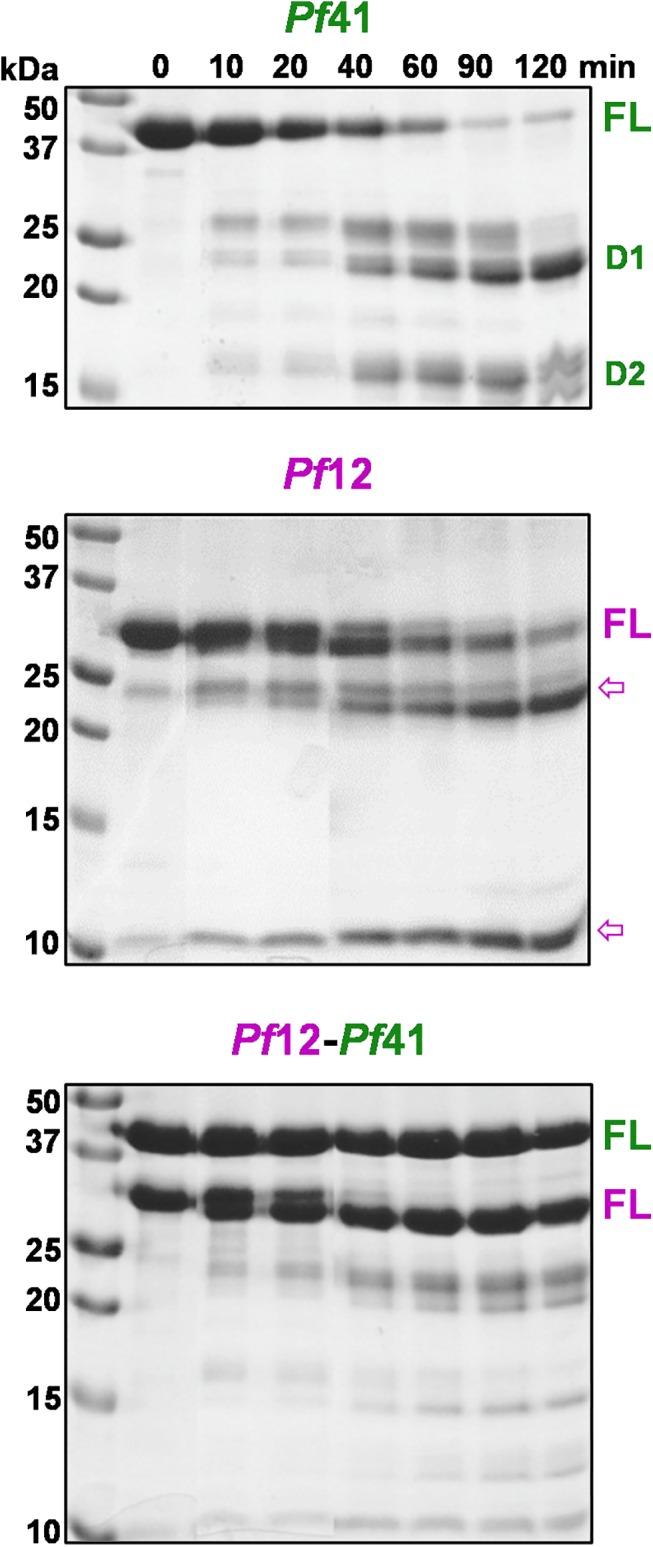
Trypsin protection assay reveals complex-dependent proteolytic resistance. SDS-PAGE analysis under reducing conditions of trypsin cleavage over time of *Pf*41 (top), *Pf*12 (middle) and *Pf*12-*Pf*41 mixture (bottom). FL, full length. Expected molecular weights: *Pf*41 FL, 41 kDa; D1, 25 kDa; D1 with the ID clipped in predicted coil region, 18 to 23 kDa; D2, 16 kDa. *Pf*12 FL, 34 kDa (C-term clipped: 32 kDa); D1, 18 kDa; D2, 16 kDa; N-term/C-term fragments from disordered loop cleavage: 10/24 kDa. *Pf*41 D1 doublet (+/- ID) and D2 were confirmed by mass spectrometry. Magenta arrows indicate clear *Pf*12 cleavage products.

While it is evident that the *Pf*41 ID is necessary for binding *Pf*12, the underlying mechanism by which it promotes assembly is unclear. For example, the *Pf*41 ID could directly engage *Pf*12, it could serve an indirect role by optimizing the orientation of the *Pf*41 6-Cys domains, or it could promote heterodimer assembly through a combination of indirect and direct mechanisms. We reasoned that a direct role in binding would likely provide the labile ID with protection from proteolysis while a purely indirect function would maintain its proteolytic susceptibility. To test this, we carried out a series of trypsin protease protection assays (Fig 4). *Pf*41 was approximately 50% degraded by trypsin after 20 minutes of incubation at 4°C; degradation bands were confirmed by mass spectrometry to be D2 and two forms of D1, the smaller of which lacks β9 and a portion of the ID (Fig 4, top). The observed degradation pattern fits with *Pf*41 having an exposed trypsin cleavage site immediately preceding the D1-D2 linker as well as several potential cleavage sites within the second half of the ID that is predicted to be largely void of stabilizing secondary structure elements (Fig 1E). Accessibility of the *Pf*41 linker is also consistent with the conformational flexibility of D1 and D2 enabled by similar linkers in the *T*. *gondii* SRS proteins [31, 32]. *Pf*12 was marginally more stable than *Pf*41 with approximately 50% degraded after 40 minutes (Fig 4, middle). The two predominant *Pf*12 degradation products likely correspond to cleavage in the β4-β6 loop of D1 that was disordered in the *Pf*12 crystal structure [15], resulting in 10 kDa and 24 kDa products. The doublet for *Pf*12 that appears shortly after trypsin addition likely represents cleavage of the exposed and disordered C-terminal tail. In stark contrast, proteolysis of the *Pf*12-*Pf*41 heterodimer with trypsin was substantially reduced even after 120 minutes (Fig 4, bottom). These data support a model where the *Pf*41 ID becomes more tightly organized upon complex formation, likely through directly mediating complex formation with *Pf*12. Notably, a previous study using western blots of reduced protein samples extracted from schizont and merozoite stage parasites showed that only one *Pf*41 protein band was observed [14]. Thus, complex formation with *Pf*12 appears to protect the *Pf*41 ID from degradation both in an *in vitro* and a biological context.

## Discussion

The 6-Cys s48/45 surface antigens are differentially presented on all life cycle stages of *Plasmodium* species suggesting important roles in enabling these parasites to interact with their environment. While a recent study provided intriguing insight into the role of *Pf*s47 in modulating the immune response to *P*. *falciparum* in the mosquito [12], detailed functional profiles of the human blood-stage merozoite expressed family members have been elusive. This is especially true for *Pf*12 and *Pf*41 where recent knockout studies showed no clear phenotype, although a complicating factor in interpreting these experiments may lie in the ability of parasites to adapt during the extensive time required for *in vitro* culturing [14]. Furthermore, a general lack of structural information describing both the individual 6-Cys proteins and the overall assembled *Pf*12-*Pf*41 heterodimeric complex has limited mechanistic insight.

The structural characterization of *Pf*12 reported in 2013 provided the first detailed architectural insight into tandem 6-Cys domains [15]. With respect to *Pf*41, however, an additional 120 residues predicted to be inserted as a spacer between the two 6-Cys domains have complicated *Pf*41 modeling efforts and led to ambiguity in defining its assembly with *Pf*12. Towards resolving this uncertainty, we first determined the structure of *Pf*41. Strikingly, structural analysis revealed that the additional residues in *Pf*41 map to three distinct regions: a large inserted domain-like region (ID) of 108 residues (Thr117 to Ile225) between the β8 and β9 strands of the D1 core that is largely proteolyzed in the final structure, the D1 β9 strand, and the short inter-domain linker (Fig 1). The observation that the newly identified ID within D1 appears to be unique to *Pf*41 suggests a *Pf*41-specific function that we reasoned involved coordination of *Pf*12. To test this, we engineered a form of *Pf*41 where the ID was replaced by a short glycine-serine linker and showed by ITC that binding to *Pf*12 was abrogated (Fig 3). While these data showed the *Pf*41 ID is necessary to coordinate *Pf*12 with high affinity, we were unable to show sufficiency due to poor stability of the ID in the absence of both 6-Cys domains. We then used a protease protection assay to investigate the mechanism by which the ID promotes *Pf*12-*Pf*41 complex formation. These data showed that the proteolytic susceptibility of the ID was nearly eliminated in the context of *Pf*12 consistent with a direct role in coordinating *Pf*12 (Fig 4).

**Fig 5:**
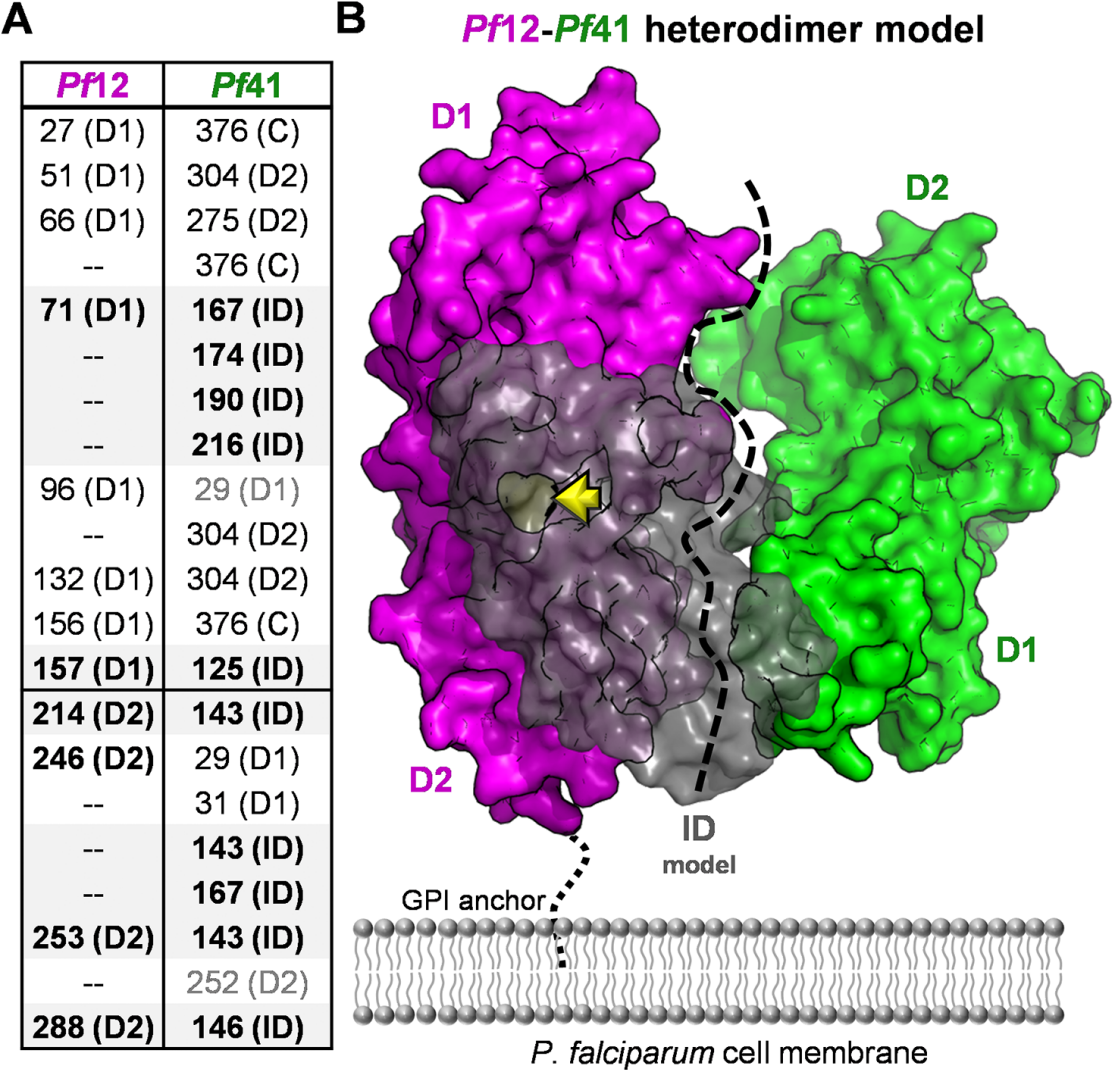
A refined *Pf*12-*Pf*41 heterodimeric assembly model. **(A)** Cross-linking designations revised from [15]; *Pf*41 ID specific interactions are bolded and shaded light grey. **(B)** Refined model of *Pf*12-*Pf*41 complex generated by manual docking and guided by cross-linking data. *Pf*12 is shown as a magenta surface, and *Pf*41 as a green surface except a model of the ID generated in iTASSER [42] displayed as a grey semi-transparent surface. The *Pf*41 ID model represents the relative size of this region compared to D1 and D2 and approximates how the ID could contact both *Pf*12 D1 and D2, but further studies probing the detailed structure and flexibility of this region are still needed. A previously identified *Pf*41 ID phosphorylation site [43] is shown in yellow and indicated by a yellow arrow. The black dotted line indicates uncertainty in the exact interface between the tandem 6-Cys domains of *Pf*12 and *Pf*41.

The structural and solution binding data reported here provided an opportunity to re-evaluate our previous *Pf*12-*Pf*41 cross-linking data [15]. Originally, cross-linked peptides were assigned to *Pf*41 D1, Linker, or D2 based on the previously predicted architecture of *Pf*41 with the spacer/linker region separating the two 6-Cys domains (Fig 1A). However, with the unambiguous domain assignments enabled by our *Pf*41 structure (Fig 3A), we can now confidently reassign the ambiguous spacer peptides to the ID, β9 of D1, or the short inter-domain linker. Thus, with our reassigned cross-link designations we obtain a more accurate model of the *Pf*12-*Pf*41 interaction: *Pf*12 is in close proximity to the *Pf*41 ID as evidenced by ten crosslinks to this region, while cross-links between *Pf*12 and *Pf*41 D1 (three), D2 (five), or the C-terminal tail (three) were much fewer (Fig 5A), which is consistent with the ITC and protease protection data (Figs 3 and 4). While it is possible that the *Pf*41 ID solely contributes to the binding interface between *Pf*41 and *Pf*12, the cross-linking data combined with polymorphism analyses of *Pf*12 and *Pf*41 homologs are consistent with at least weak interactions between the 6-Cys domains of *Pf*41 and *Pf*12 to form an antiparallel interface [40, 41]. Moreover, our trypsin protease protection assay indicates that the *Pf*41 inter-domain linker (RSNNNVI) is stabilized in the presence of *Pf*12, indicating a tightening of the *Pf*41 6-Cys domains upon complex formation with *Pf*12 (Fig 4). Based on these data, we propose a refined model of the *Pf*12-*Pf*41 heterodimer, where the *Pf*41 ID serves as the key structural bridge to anchor the complex (Fig 5B).

While the *Pf*41 ID is clearly a critical component of the *Pf*12-*Pf*41 complex assembly, the ID may endow *Pf*41 with multi-functional capabilities as its extended size appears to be substantially over-engineered for simply mediating the interaction with *Pf*12. This is particularly evident when analyzed in the context of the SRS29B and SRS16C proteins from *T*. *gondii* where the analogous SRS domains are sufficient for homodimerization [31, 33]. Notably, however, these SRS proteins are tethered to the membrane via a GPI anchor thereby minimizing the entropic penalty that must be overcome compared to soluble *Pf*41 binding a membrane-anchored protein. One possibility for an additional role for the *Pf*41 ID comes from a previous study that found that in parasites with *Pf*12 genetically deleted, a small amount of *Pf*41 was observed on the parasite surface suggesting that *Pf*41 is able to bind at least one other merozoite membrane-anchored molecule [14]. It is thus tempting to speculate that the proteolytic susceptibility and lack of predicted secondary structure elements within the ID, particularly in the second half, may indicate a level of flexibility that enables the ID to bind other molecules with variable induced fit depending on the partner [37]. Moreover, the binding of *Pf*41 to *Pf*12, or other parasite membrane proteins or even host derived partners, could be enhanced and/or enabled by post-translational modifications, such as the phosphorylation of *Pf*41 ID Ser137 [43] (Fig 5B, yellow arrow). Whether or not the *Pf*41 ID has functions additional to its role in *Pf*12 coordination, the importance of the *Pf*41 ID is supported by a previous study showing that antibodies in human immune sera specifically recognize this region [17].

## Conclusions

*Pf*12 and *Pf*41 form a stable heterodimer on the surface of the infective *Plasmodium* merozoite, but the function of this complex remains elusive [14]. In the absence of a definitive biological role, however, the biophysical studies of *Pf*12 [15] and *Pf*41 (here) reveal important insight into the unique architectural features of the individual proteins and help unravel key mechanistic details underpinning assembly of the heterodimer. In particular, the identification and characterization of the ID in *Pf*41 as the crucial region enabling coordination to *Pf*12 has allowed us to refine the model of the *Pf*12-*Pf*41 complex on the parasite surface. As genetic tools continue to improve it will be important to revisit the function of the *Pf*12-*Pf*41 complex and assess what role, if any, the *Pf*41 ID plays in directly interfacing with the host.
